# Synergistic Effect of Strontium Doping and Surfactant Addition in Mesoporous Bioactive Glass for Enhanced Osteogenic Bioactivity and Advanced Bone Regeneration

**DOI:** 10.3390/polym17020187

**Published:** 2025-01-14

**Authors:** Ya-Yi Chen, Tien-Li Ma, Pei-Jung Chang, Yuh-Jing Chiou, Wei-Min Chang, Ci-Fen Weng, Chin-Yi Chen, Yu-Kang Chang, Chung-Kwei Lin

**Affiliations:** 1 Department of Stomatology, Tung’s Taichung Metro Harbor Hospital, Taichung 435, Taiwan; yayichen2013@gmail.com; 2 Doctoral Program in Medical Biotechnology, National Chung Hsing University, Taichung 402, Taiwan; 3 Research Center of Digital Oral Science and Technology, College of Oral Medicine, Taipei Medical University, Taipei 110, Taiwan; peronchang@tmu.edu.tw (P.-J.C.); chiou@gm.ttu.edu.tw (Y.-J.C.); weiminchang@tmu.edu.tw (W.-M.C.); chencyi@fcu.edu.tw (C.-Y.C.); 4 School of Dental Technology, College of Oral Medicine, Taipei Medical University, Taipei 110, Taiwan; tlma113005@tmu.edu.tw; 5 Graduate Institute of Manufacturing Technology, National Taipei University of Technology, Taipei 106, Taiwan; 6 Department of Chemical Engineering and Biotechnology, Tatung University, Taipei 104, Taiwan; 7 School of Oral Hygiene, College of Oral Medicine, Taipei Medical University, Taipei 110, Taiwan; 8 Department of Materials Science and Engineering, Feng Chia University, Taichung 407, Taiwan; nina20030@gmail.com; 9 Department of Medical Research, Tung’s Taichung Metro Harbor Hospital, Taichung 435, Taiwan; 10 Department of Post-Baccalaureate Medicine, College of Medicine, National Chung Hsing University, Taichung 402, Taiwan; 11 Department of Nursing, Jenteh Junior College of Medicine, Nursing and Management, Miaoli 356, Taiwan

**Keywords:** mesoporous bioactive glass, hydroxyapatite, strontium, P123, bone tissue engineering

## Abstract

Mesoporous bioactive glass (MBG) is an advanced biomaterial widely recognized for its application in bone regenerative engineering. This study synthesized an MBG powder (80 mol% SiO_2_, 5 mol% P_2_O_5_, and 15 mol% CaO) using a facile sol-gel method with the non-ionic surfactant Pluronic^®^ P123, which acted as a pore-forming agent. MBGs form bioactive surfaces that facilitate HA formation, and the presence of Pluronic^®^ P123 increases the surface area and promotes HA nucleation. Various percentages of strontium (Sr) doping were examined to improve bioreactivity, biological response, and bone formation, with 3SMBG (3 mol% Sr) showing the highest specific surface area. In vitro biocompatibility tests revealed HA formation on all glass surfaces after immersion in simulated body fluid (SBF), indicated by sheet-like HA morphologies, the presence of PO_4_^3−^ and CO_3_^2−^ functional groups, and the amorphous structure along with SrCO_3_ crystalline phases corresponding to HA and Sr-HA structures. Sr doping resulted in delayed initial degradation and sustained release of Sr^2+^, achieving over 95% cell viability. Surfactant-induced mesoporous structure and Sr incorporation synergistically enhance osteocyte induction and formation in vitro. These findings suggest that Sr-doped MBG, particularly with P123-assisted Sr/Ca substitution, optimizes the material’s properties for advanced bone regenerative applications.

## 1. Introduction

Mesoporous bioactive glasses (MBGs) with ordered, interconnected mesopores (5–20 nm) in the SiO_2_-P_2_O_5_-CaO system exhibit higher specific surface area and pore volume than non-MBGs [1,2]. MBGs show excellent cell compatibility and bioactivity, making them promising for tissue engineering, drug delivery, and cancer treatment [3,4]. Degradation of MBG raises pH, releasing calcium and phosphate ions to promote HA formation and facilitate tissue repair and regeneration [5].

Successful MBG synthesis relies on surfactants like cetyltrimethylammonium bromide (CTAB), Pluronic^®^ P123, enabling stable frameworks with tunable nanoscale pore sizes and abundant surface silanol groups [6]. Silica-based BG shows superior bone regeneration potential over HA and promotes angiogenesis through vascular endothelial growth factor (VEGF) production [7]. MBGs’ multifunctionality allows for therapeutic ion incorporation and surface functionalization without compromising bioactivity or drug delivery, enhancing their biomedical applications [8]. Strontium (Sr) is favored for its osteogenic properties, stimulating osteogenic differentiation, inhibiting adipogenesis, and reducing osteoblast apoptosis [9,10]. Sr enhances marrow stromal cells (MSCs) response and suppresses osteoclast differentiation by inhibiting receptor activator of nuclear factor-κB ligand (RANKL) [11,12]. Localized Sr^2+^ release using Sr-doped bone graft substitutes is a promising strategy in bone tissue engineering [13].

In the context of MBGs, incorporating Sr into the scaffolds has been found to enhance new bone formation in osteoporotic bone defects [14]. Moreover, the controlled release of Sr^2+^ from Sr-MBG scaffolds facilitates local bone formation while inhibiting bone resorption [15,16]. The high Sr content can influence the mesoporous structure and surface area of MBGs, impacting their overall morphology. Furthermore, Sr-MBG scaffolds effectively released Sr ions, enhancing alkaline phosphatase (ALP) activity and osteogenic gene expression in periodontal ligament cells (PDLCs) [17]. Kim et al. showed that Sr-MBG nanoparticles enhance bone and dentin regeneration by co-delivering Sr^2+^ and phenanthroline, increasing gene expression and mineralization [18]. A study synthesized mesoporous glasses doped with 1, 3, and 5 mol% Sr^2+^ in the P_2_O_5_-CaO-Na_2_O system using sol-gel method with P123 as a template, demonstrating enhanced bioactivity of Sr-doped mesoporous glasses through increased cell proliferation and ALP activity in human osteosarcoma cells, highlighting their potential in promoting bone formation [19]. Newman et al. further validated the efficacy of Sr-doped silicate-based glass coatings applied to roughened Ti6Al4V in promoting rabbit osteoblast differentiation and proliferation [20].

Studies have indicated that an MBG with 80 mol% SiO_2_, 5 mol% P_2_O_5_, and 15 mol% CaO composition demonstrates optimal bioactivity [21]. In this study, we focus on synthesizing mesoporous glasses doped with varying concentrations (1, 2, 3, and 5 mol%) of Sr^2+^ in the P_2_O_5_-CaO-SiO_2_ system using a sol-gel method with the nonionic triblock copolymer P123 as a template agent. P123 was chosen for its ability to create larger, stable mesoporous structures over various pH levels and temperatures [22], due to its lower toxicity than CTAB, making it ideal for biomedical applications [23]. The study systematically evaluates the bioactivity and dissolution behavior of Sr-doped MBGs, focusing on hydroxyapatite (HA) formation and ion release. Advanced characterization techniques were employed to investigate structural changes, functional group modifications, and ion exchange dynamics, providing insights into the synergistic effects of Sr doping and the mesoporous structure on bone regenerative potential. Lastly, the biocompatibility of the optimized Sr-doped MBG and non-doped MBG was thoroughly assessed. The use of Pluronic^®^ P123 as a polymeric template for MBGs is well-established; however, the effects of Sr doping levels on bioactivity and structural stability remain underexplored. This study systematically examines Sr doping (1–5 mol%), identifying 3 mol% as optimal for balancing HA formation and mesopore stability. Our findings provide new insights into the relationship between Sr content and bioactive performance, addressing a gap in the literature. Building on prior studies, such as Vallet-Regí et al. [24,25], we highlight how polymer-driven templating and Sr doping synergistically enhance HA nucleation and ion release kinetics. These results advance the design of Sr-doped MBGs for bone regeneration, demonstrating their potential to optimize bioactivity while preserving structural integrity.

## 2. Materials and Methods

### 2.1. Preparation of Strontium-Doped Mesoporous Bioactive Glass (SMBG) Powder

The chemicals used for preparation of SMBG are tetraethyl orthosilicate (TEOS; Alfa Aesar, Ward Hill, MA, USA), triethyl phosphate (TEP; Acros, Geel, Belgium), calcium nitrate tetrahydrate (Alfa Aesar, Ward Hill, MA, USA); strontium nitrate (Sigma Aldrich, St. Louis, MO, USA), poly ((ethylene oxide)-block-poly (propylene oxide)-block-poly (ethylene oxide)) (PEO-PPO-PEO; Pluronic^®^ P123; Sigma Aldrich, St. Louis, MO, USA; M_n_ = 5800), hydrochloric acid (Showa, Tokyo, Japan).

**Figure 1:**
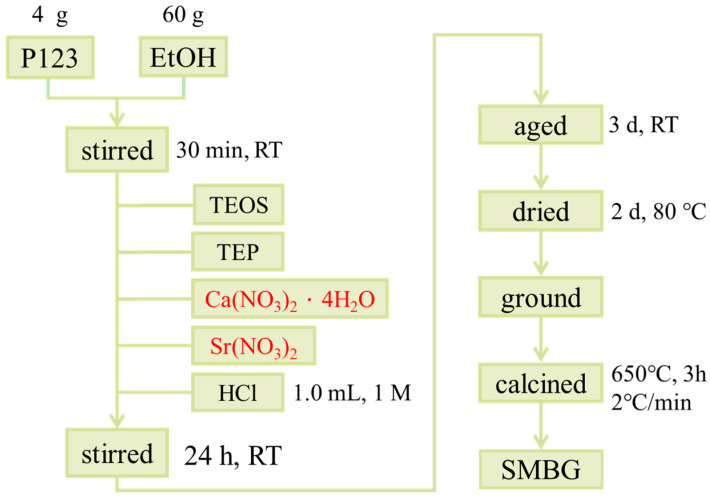
Experimental flow chart of the preparation of MBG.

Pluronic^®^ P123 was blended with alcohol and stirred for 30 min until completely dissolved. Following that, TEOS, TEP, calcium nitrate tetrahydrate, strontium nitrate, and 1M hydrochloric acid were sequentially added at 45-min intervals. The strontium nitrate was added at molar proportions of 1%, 2%, 3%, and 5%. Strontium nitrate was used as the strontium precursor during the sol-gel synthesis of MBG. Sr ions were incorporated into the silica network as network modifiers, partially replacing Ca ions, which modulates the glass structure and enhances its bioactivity. The mixture was continuously stirred for a duration of 24 h, followed by a three-day aging process, and subsequently subjected to a two-day drying process at 80 °C to facilitate solvent evaporation and subsequent pulverization, resulting in the formation of powdered specimens. Finally, the specimens were heated at a controlled rate of 2 °C/min, reaching a final temperature of 650 °C, and maintained at this temperature for a period of three hours to ensure the complete elimination of surfactants and impurities, thereby yielding specimens labeled as 0SMBG, 1SMBG, 2SMBG, 3SMBG, and 5SMBG according to their respective Sr doping content. The experimental procedure is shown in Figure 1. The composition and precursors of different MBGs are shown in Table 1.

### 2.2. Characterization of SMBG Powder

#### 2.2.1. Specific Surface Area and Pore Size Distribution

The measurement of surface area using the Brunauer–Emmett–Teller (BET) method is based on gas adsorption theory at solid surfaces. As the pressure increases, the amount of gas molecules adsorbed and the relative pressure (P/P0) also increase. Therefore, the gas adsorption at each equilibrium pressure was recorded, and the BET theory model was used to calculate the surface area. The adsorption/desorption isotherm curve illustrates the surface area and surface and internal pore characteristics of the material. The instruments used were the ASAP 2020 from Micromeritics (Norcross, GA, USA) and SA-9600 from HORIBA (Irvine, CA, USA).

#### 2.2.2. Surface Morphology of MBG

A field emission scanning electron microscope (FE-SEM) was used to examine the surface morphology and structure of SMBG powders. Energy dispersive X-ray spectroscopy (EDS) was employed for elemental analysis by comparing the intensity of different elemental spectral lines. For SEM imaging, the sample was dispersed in anhydrous alcohol, dropped onto a copper grid, dried, and coated with platinum (Pt) for enhanced conductivity. Silk fibers were directly attached to carbon tape and also coated with Pt. The FE-SEM used was an S-4800 from HITACHI (Tokyo, Japan). The surface morphology and particle dimensions of the MBG powders were analyzed using SEM. The particles exhibited dimensions ranging from 100 to 300 μm.

#### 2.2.3. Characterization of MBG Structural Phases

The X-ray diffraction (XRD) analysis was used to confirm that the synthesized MBG powders were amorphous before immersion in simulated body fluid (SBF), as indicated by the broad characteristic peaks. The XRD peaks’ 2θ positions and intensities were determined using X’pert Highscore software (version 5.2) to identify the material’s structure. The material’s diffraction peaks were compared with ICDD card numbers and plotted using Origin software to characterize the composition, phase composition, and structure of different parameter MBG powders. The instrument used is a D8 Discover, Bruker (Billerica, MA, USA). (Parameters: 45 kV, 25 mA, scanning rate 2 °C/min, scanning range of 20° to 80°).

#### 2.2.4. Chemical Bonding and Functional Groups

Fourier-transform infrared spectroscopy (FT-IR) was used to identify the specific functional groups and chemical bonding within the sample. The FT-IR analysis was performed using the FT-IR Spectrometer Frontier, manufactured by PerkinElmer (Waltham, MA, USA), which was equipped with an attenuated total reflectance (ATR) accessory. The spectral range for wavenumber scanning spanned from 500 cm^−1^ to 4000 cm^−1^.

#### 2.2.5. Analysis of Ion Concentration Release

The quantification of trace element concentrations in the samples was accomplished through the utilization of inductively coupled plasma optical emission spectrometry (ICP-OES). The sample solution was nebulized and introduced into the inductively coupled plasma, where the elevated temperature and energy facilitated the ionization of the atomized gas. Subsequently, upon subjecting the ionized atoms to higher temperatures, they transitioned from their transient excited states to the more stable ground state, and during this process, each element emitted characteristic spectral lines. Semi-quantitative analysis was conducted using an electron detector to elucidate the variations in ion concentration within the samples subsequent to their immersion in simulated body fluid (SBF) for distinct time intervals.

The chemicals used for SBF preparation were sodium chloride (NaCl; Uni region, New Taipei City, Taiwan), sodium bicarbonate (NaHCO_3_; Sigma Aldrich, St. Louis, MO, USA), potassium chloride (KCl; J.T.Baker, Radnor, PA, USA), potassium phosphate dibasic (K_2_HPO_4_·3H_2_O; Sigma Aldrich, St. Louis, MO, USA), magnesium chloride hexahydrate (MgCl_2_·6H_2_O; Sigma Aldrich, St. Louis, MO, USA), sodium sulfate (Na_2_SO_4_; Merck, Darmstadt, Germany), Tris(hydroxymethyl)aminomethane (Tris; (HOCH_2_)_3_CNH_2_; Sigma Aldrich, St. Louis, MO, USA), and hydrochloric acid (HCl; Showa, Tokyo, Japan). The instrument used was an OPTIMA 2000 DV, Perkin Elmer (Waltham, MA, USA), and Sr was the analyzed element, with concentration reported in ppm.

### 2.3. In Vitro Mineralization Test

SBF, which mimics the ionic concentration of human blood plasma, was used to immerse samples with different parameters to obtain the formation of surface apatite and characterize their bioactivity. The preparation of the SBF in this study was based on the method proposed by Dr. Kokubo’s team [26]. Precisely weighed 200 mg of sample powder was immersed in 80 mL of SBF, and the container material was polypropylene (PP). The immersion was carried out at 37 °C and 60 rpm for 1, 3, and 7 days in a constant temperature water bath. After centrifugal washing with deionized water and alcohol, the powder was dried at 80 °C for 24 h, and the weight change was recorded after accurate weighing. The surface morphology was observed by SEM, and the structural composition and chemical bond changes were analyzed by XRD and FT-IR.

### 2.4. Biocompatibility Test

In this study, the Alamar Blue assay was used to analyze the samples. The enzyme-linked immunosorbent assay (ELISA) was used to detect the absorbance values at 570 nm, representing mitochondrial metabolic activity. Human osteosarcoma cells (MG63, Bioresource Collection and Research Center (BCRC) 60279, Hsinchu, Taiwan) and mouse fibroblast cells (L929, Bioresource Collection and Research Center (BCRC) RM60091, Hsinchu, Taiwan) were selected as test cells in this study. Sterile test materials were weighed in equal amounts and extracted with serum-free cell culture medium at 37 °C for 24 h to prepare the material extract. Test cells were seeded at a density of 1 × 10^4^ cells per well in a 96-well plate and allowed to adhere overnight. The next day, the extract was added to the cells and treated at different dilution ratios. After 24 h of incubation, Alamar Blue was added and the cell viability was detected by measuring the absorbance at 570 nm. Statistical analysis was conducted using the Kruskal–Wallis H test to compare differences in cell viability between groups.

## 3. Results and Discussion

### 3.1. Characteristics of Strontium-Doped Mesoporous Bioactive Glass (SMBG) Powder

#### 3.1.1. XRD Analysis of SMBG Powder

**Figure 2:**
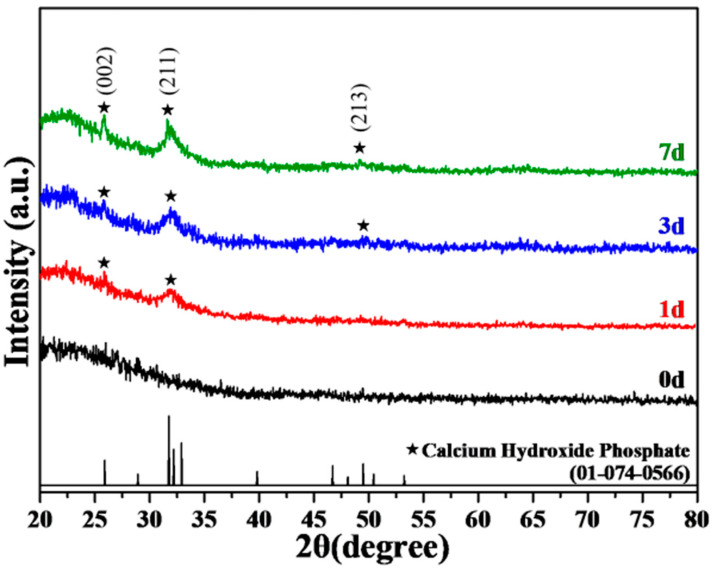
XRD patterns of 0SMBG before and after immersion in SBF for 1, 3, and 7 days.

Figure 2 shows the XRD analysis results of 0SMBG before and after immersion in SBF for 1, 3, and 7 days. Before immersion, no distinct peaks were observed except for a broad peak between 20° and 40°, indicating an amorphous structure of the powder. After immersion in SBF, characteristic peaks appeared, which matched well with the peaks of hydroxyapatite (PDF 01-074-0566). After 1 day of immersion, the peaks at 25.8° and 31.7° corresponded to (002) and (211) planes, respectively. After 3 days of immersion, the diffraction peaks became narrower and stronger, with the appearance of a new peak at 49.4° corresponding to the (213) plane. After 7 days of immersion, the intensity of the characteristic peaks further increased, confirming the formation of the HA structure of 0SMBG powder after immersion in SBF solution, and the crystallinity increased with the immersion time. The 0SMBG was amorphous before SBF immersion. After immersion, hydroxyapatite peaks appeared at 25.8°, 31.7°, and 49.4°, with increasing intensity and crystallinity over 1, 3, and 7 days.

**Figure 3:**
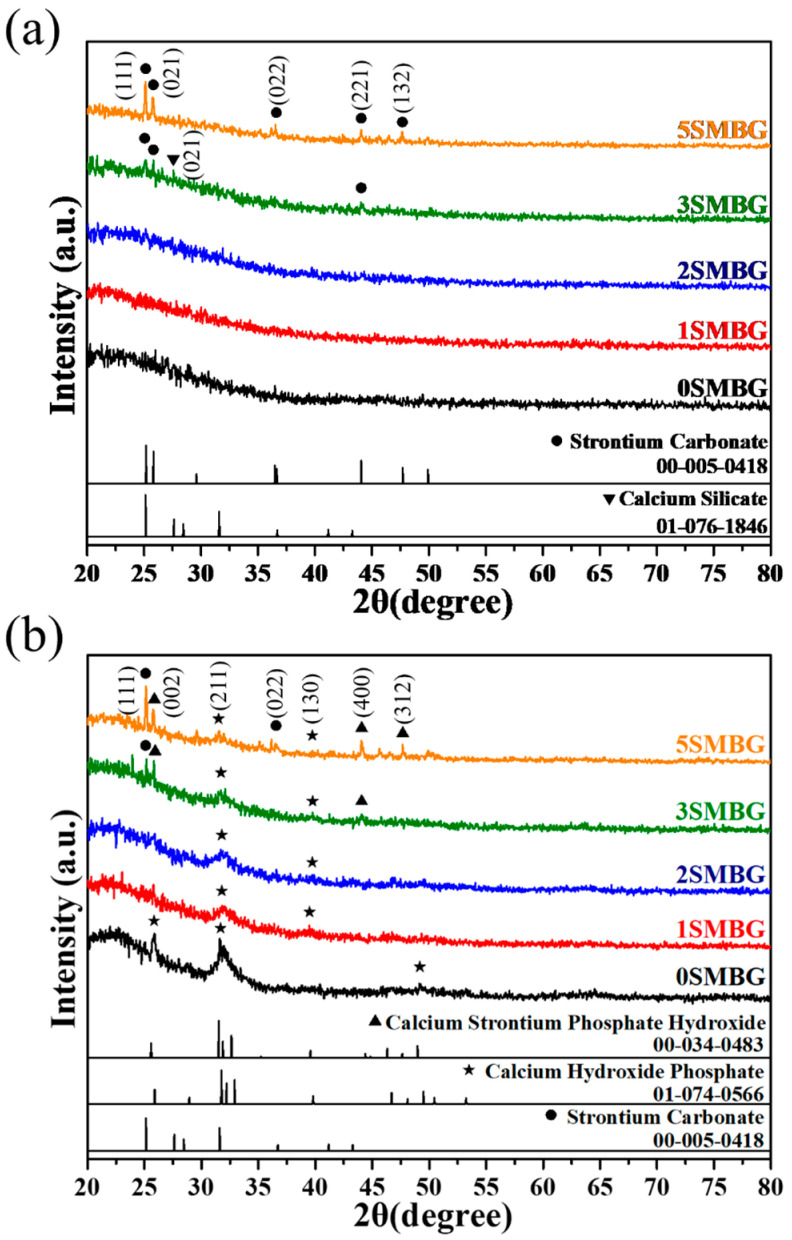
XRD patterns of 0S-5SMBG before (**a**) and after (**b**) soaking in SBF for 7 days.

Figure 3 presents the XRD analysis of 0S-5SMBG powders before and after immersion in SBF. As shown in Figure 3a, the XRD patterns of 1SMBG, 2SMBG, and undoped 0SMBG exhibit an amorphous structure, similar to that of the powder before immersion in SBF. In contrast, 3SMBG and 5SMBG doped with higher amounts of Sr exhibit diffraction peaks at 25.1° and 25.8°, corresponding to the (111) and (021) crystal planes of strontium carbonate (SrCO_3_), respectively. Moreover, 5SMBG shows characteristic peaks of SrCO_3_ at 36.6°, 44.1°, and 47.6°, corresponding to the (022), (221), and (132) crystal planes, respectively, which are consistent with the strontium nitrate (PDF 00-005-0418). In addition, a characteristic peak of wollastonite (Ca_3_(Si_3_O_9_)) appears at 2θ = 27.6° on the XRD pattern of 3SMBG, corresponding to the (021) crystal plane and consistent with the PDF 01-076-1846. The mechanism underlying the formation of Ca_3_(Si_3_O_9_) is not yet fully understood, but it is believed to be related to the substitution of Ca by Sr in the hydrolysis and condensation process, which affects the internal structure of MBG. In comparison to standard hydroxyapatite (PDF 01-074-0566), characteristic peaks at 31.7° shifted slightly to 32.0°, indicating lattice expansion caused by Sr^2+^ substituting Ca^2+^. The observed peaks at 25.5° and 44.1°are associated with the crystalline phases of Sr-doped hydroxyapatite (Sr-HA). These peaks confirm the successful incorporation of Sr into the hydroxyapatite lattice during the immersion process, consistent with previous studies on Sr-doped bioactive glasses.

Moreover, P123, which acts as a pore-forming agent, also participates in the internal reactions, causing the appearance of wollastonite instead of calcium silicate (Ca_2_SiO_4_), which would normally be detected. Some studies suggest that MBG doped with P123 has a slightly different calcium carbonate structure [27]. Additionally, Wu et al.’s study showed that the strontium ion (Sr^2+^: 0.116 nm) has a slightly larger radius than the calcium ion (Ca^2+^: 0.094 nm), which causes the glass network to become looser and expand, lowering the activation energy for crystallization. Furthermore, the reaction between Sr and P123-generated carbides during the annealing process leads to the formation of SrCO_3_ [28], proving the presence of Sr doping in MBG.

All powders produced HA, exhibiting characteristic peaks at diffraction angles of 31.7° and 39.7° corresponding to (211) and (130) crystal planes, respectively, as shown in Figure 3b. The original Ca_3_(Si_3_O_9_) structure in 3SMBG disappeared after being soaked in SBF for 7 days. The characteristic peaks of the SrCO_3_ structure in 3SMBG and 5SMBG, except for the (111) crystal plane at 25.1°, transformed into the strontium hydroxyapatite (Ca_8_Sr_2_(PO_4_)_6_(OH)_2_) structure at other positions, with characteristic peaks at 25.5°, 43.9°, and 47.6° corresponding to (002), (400), and (312) crystal planes, respectively. The characteristic peaks of HA and Ca_8_Sr_2_(PO_4_)_6_(OH)_2_ matched with PDF 00-074-0566 and 01-034-0483, respectively, indicating that all four Sr-doped SMBGs exhibited bioactivity and could form the HA structure after immersion in SBF.

#### 3.1.2. Analysis of Specific Surface Area of SMBG Powder

**Figure 4:**
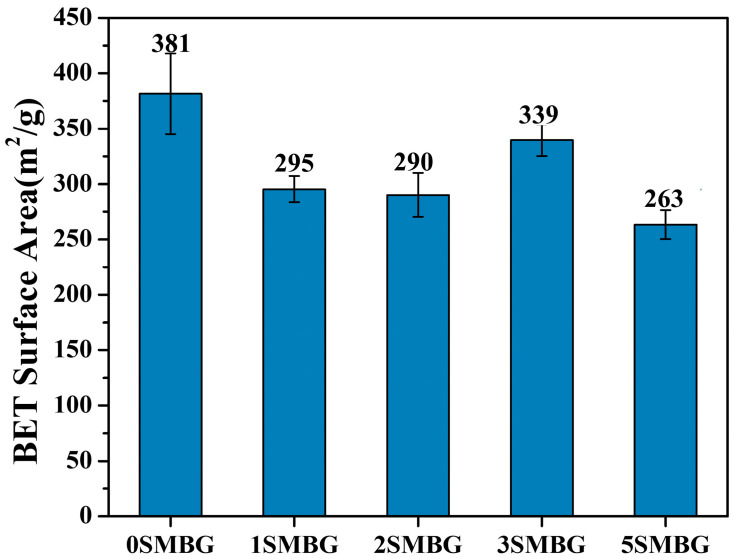
BET surface area analysis of 0SMBG-5SMBG powders.

The BET analysis results of 0S-5SMBG powders are shown in Figure 4, indicating good specific surface area values for all samples. A higher specific surface area may indicate better bioactivity. Pure MBG and various Sr-doped MBG powders were compared, and 0SMBG showed the highest value of 381 m^2^/g. As the Sr content increased, the specific surface area and pore volume decreased [29], with values of 295, 290, 339, and 263 m^2^/g for 1, 2, 3, and 5 mol% Sr doping, respectively. Among these, 3SMBG exhibited the best performance compared to other SMBGs. The Sr^2+^ was incorporated into the silica network as a network modifier, replacing smaller Ca^2+^ and causing some loss of structural properties, resulting in thicker silica lattice, lower density, and reduced mesopores [30]. As a network modifier, Sr^2+^ replaced smaller Ca^2+^ ions, causing changes in structural properties, including a thicker silica lattice, reduced mesoporosity, and lower density. These effects were consistent with observed shifts in XRD and FT-IR spectra.

#### 3.1.3. SEM and EDS Analysis of SMBG Powder

**Figure 5:**
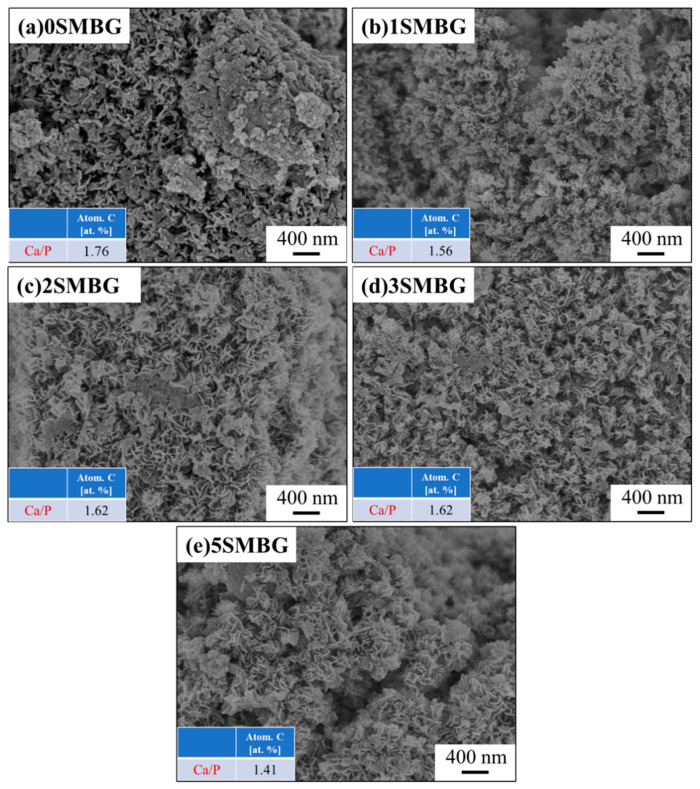
SEM morphologies and Ca/P ratios of 0S-5SMBG powders after soaking in SBF for 7 days; (**a**) 0SMBG, (**b**) 1SMBG, (**c**) 2SMBG, (**d**) 3SMBG, and (**e**) 5SMBG.

The SEM analysis presented in Figure 5 reveals the changes of 0S-5SMBG immersed in SBF for seven days. After immersion, all 0S-5SMBG samples show the formation of dense, small, sheet-like structures on the surface, which is a morphology representative of HA structures [31]. This confirms that the surface of MBG is indeed dissolved and then transformed into HA. Elemental analysis by EDS gives Ca/P ratios ranging from 1.41 to 1.76, which are close to the Ca/P ratio of 1.67 in HA. It is observed that the Ca/P ratio of 5SMBG is lower than that of the other Sr-doped MBG samples due to the excessive amount of Sr, which slows down the transformation into HA.

**Figure 6:**
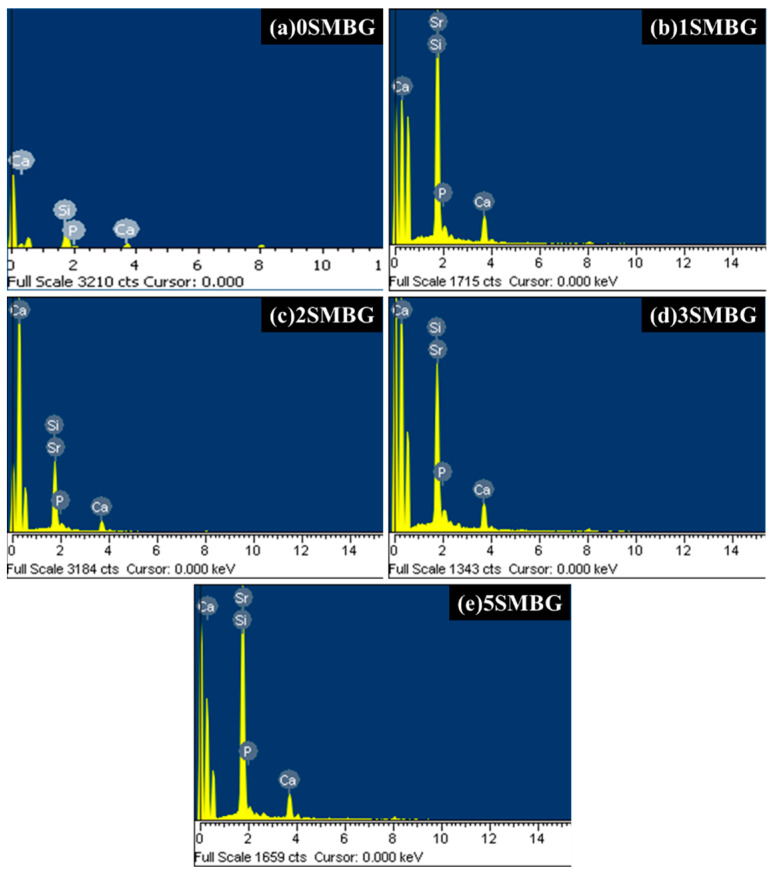
EDS analysis results of SMBG powders; (**a**) 0SMBG, (**b**) 1SMBG, (**c**) 2SMBG, (**d**) 3SMBG, and (**e**) 5SMBG.

Through the EDS analysis of 0S-5SMBG powders, we observed the presence of Sr in 1S, 2S, 3S, and 5SMBG samples, as shown in Figure 6 for 0SMBG, which confirms the successful preparation of MBG powders using the sol-gel method and doping with Sr. 

#### 3.1.4. FT-IR Analysis of SMBG Powder

**Figure 7:**
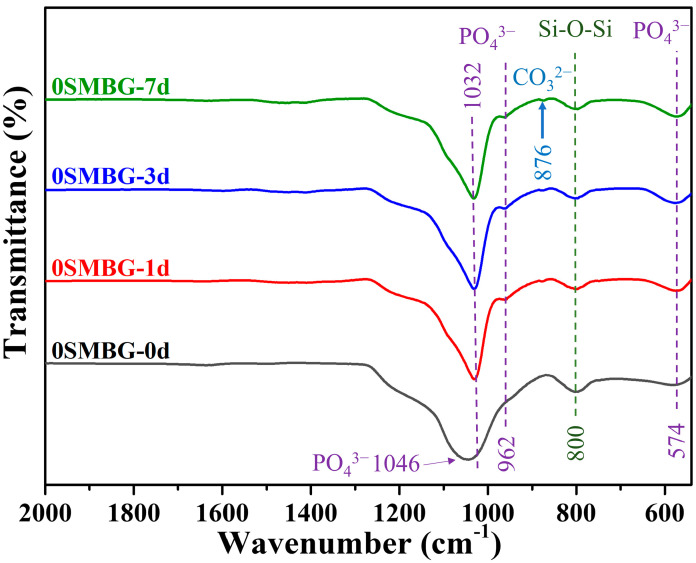
FT-IR analysis of 0SMBG before and after immersion in SBF for 1, 3, and 7 days.

The FT-IR analysis of 0SMBG before and after immersion in SBF for 1, 3, and 7 days is shown in Figure 7. The changes in functional group vibrations can be observed as the immersion time increases. In particular, the characteristic peak of the asymmetric bending vibration mode of Si-O-Si at 800 cm^−1^ becomes significantly narrower, and the characteristic peak of the asymmetric stretching mode of the phosphate group at 1046 cm^−1^ slightly shifts to 1032 cm^−1^. Additionally, symmetric stretching and bending mode characteristic peaks of PO_4_^3−^ bonds at 962 cm^−1^ and 574 cm^−1^ are newly observed. These changes indicate successful formation of HA. Notably, a small characteristic peak of the asymmetric bending vibration mode of CO_3_^2−^ at 876 cm^−1^ is observed after one day of immersion in SBF, indicating the formation of hydroxyapatite carbonate (HCA) in addition to pure HA. The absence of HCA detection by XRD is attributed to its low concentration, which does not reach its detection limit.

**Figure 8:**
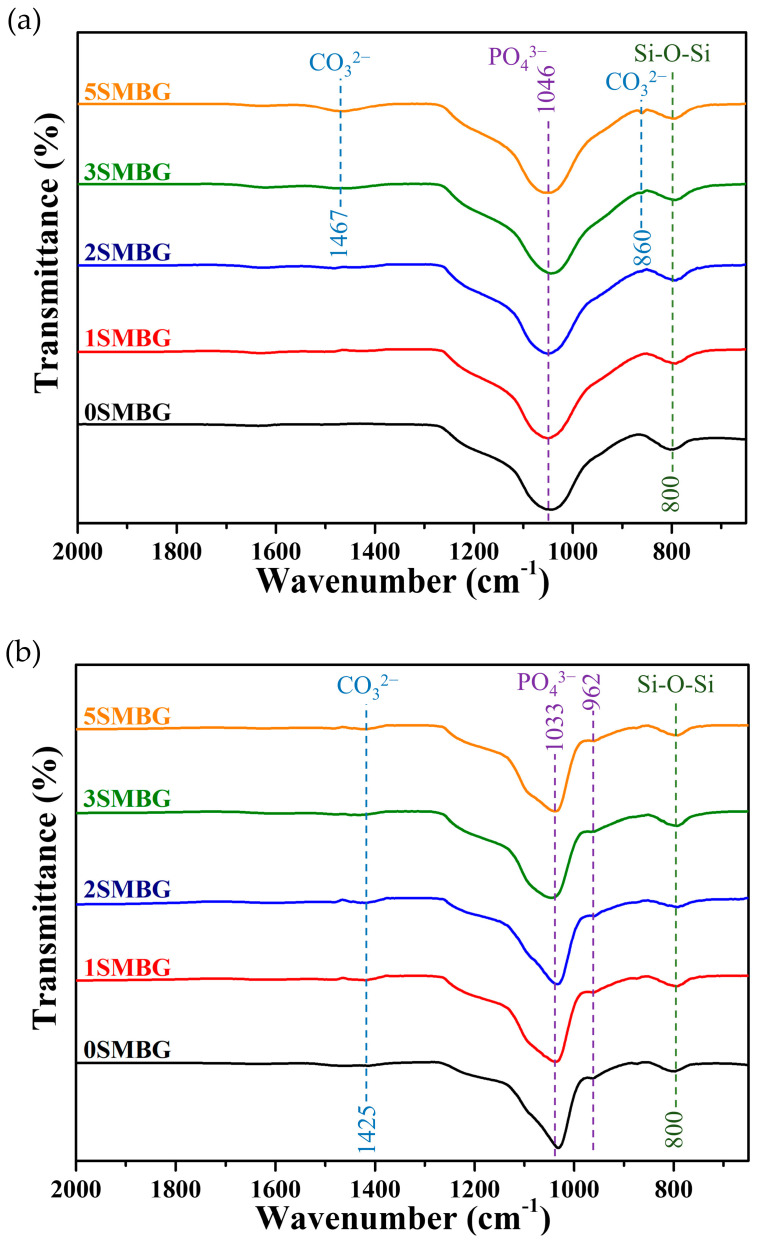
FT-IR analysis of 0S-5SMBG powders before and after soaking in SBF for 7 days, (**a**) before soaking and (**b**) after soaking.

Figure 8 shows the FT-IR analysis of 0S-5SMBG before and after soaking in SBF for 7 days. Comparing Figure 8a,b with 0SMBG in Figure 7, similar characteristic peak changes were observed after 7 days of soaking in SBF. The broad peak of the PO_4_^3−^ functional group became narrower, shifted slightly from 1046 cm^−1^ to 1033 cm^−1^, and a small peak appeared at 962 cm^−1^, indicating the formation of HA in the presence of Sr-doped MBG. In Figure 8a, CO_3_^2−^ asymmetric bending and asymmetric stretching modes were observed at 860^−1^ and 1467 cm^−1^ for 3SMBG and 5SMBG, respectively, which corresponds to the SrCO_3_ structure shown in Figure 3a. In Figure 8b, in addition to changes in the phosphate functional groups, a weak and broad asymmetric stretching peak of CO_3_^2−^ group appeared at 1425 cm^−1^ for all Sr-doped SMBGs after soaking in SBF for 7 days, indicating the exchange reaction between SrCO_3_ and SBF ions and the detection of strontium phosphate structure in XRD analysis. This confirms the formation of strontium phosphate besides pure HA. FT-IR analysis revealed shifts in PO_4_^3−^ functional group peaks (e.g., from 1033 cm^−1^ to 1028 cm^−1^), indicating structural modifications due to Sr^2+^ substitution. These shifts align with previous studies on Sr-doped hydroxyapatite and confirm the formation of Sr-HA. The FT-IR spectra were smoothed to enhance readability by reducing noise. Despite this, the characteristic peaks for PO_4_^3−^ and CO_3_^2−^ groups indicative of hydroxyapatite formation are clearly observed, consistent with the results of XRD and other analyses.

### 3.2. Post-Immersion Characterization of SMBG in SBF

#### 3.2.1. Weight Loss Analysis of SMBG Powder

Table 2 shows the weight loss of 0S-5SMBG samples before and after immersion in SBF for 1, 3, and 7 days. The initial weight of all samples was 200 mg. After one day of immersion, all samples showed a decrease in weight, with SMBG exhibiting a more significant weight loss than pure MBG. Additionally, as the Sr-doping content increased, the weight loss of 1S-3SMBG gradually decreased. This phenomenon is attributed to the deformation of the glass work caused by the addition of a small amount of Sr to MBG, which promotes network hydrolysis and increases its dissolution rate [32]. However, the Ca^2+^ ions in the structure primarily disrupt the internal connections of the network. As the content of SrO replacing CaO increases, the solubility of Ca in the structure decreases, leading to a reduction in the glass dissolution rate and weight loss. Moreover, the weight loss of 5SMBG, with the highest Sr content, was higher than that of 3SMBG, suggesting that Sr^2+^ with a larger ionic radius can break the extension of Si-O-Si bonds and the external connectivity of the network structure [33]. Therefore, more Si-O-Si bonds are broken, leading to the dissolution of the glass network during immersion in SBF.

On the third day, the weight of pure 0SMBG and 1S-3SMBG increased, indicating the formation of HA. However, the weight of 5SMBG continued to decrease due to the excess Sr^2+^ ions that hindered the initial cation exchange on the glass surface and temporarily adsorbed on the active growth sites of Ca, delaying the nucleation and precipitation rate of calcium phosphate and HA layer formation [34]. On the seventh day, the weight of pure 0SMBG and 1S-3SMBG slightly decreased, indicating that the MBG continued to degrade, causing the weight to continue to decrease. However, the weight of 5SMBG increased, indicating that HA was also generated after 7 days of immersion in SBF.

#### 3.2.2. pH Value and ICP-OES Analysis of SMBG Powder

**Figure 9:**
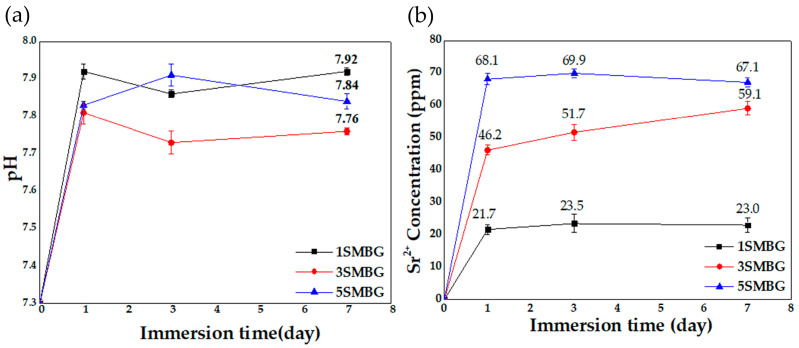
(**a**) Curves of pH values and (**b**) Sr^2+^ concentration (ICP-OES curves) of 1SMBG, 3SMBG, and 5SMBG immersed in SBF for 1, 3, and 7 days.

The pH and Sr^2+^ ion changes in SBF solution were compared among 1S, 3S, and 5SMBG with different Sr doping levels. Figure 9a presents the pH measurements of the three samples soaked in SBF solution for 1, 3, and 7 days. After one day, the pH value of 1SMBG was slightly higher than that of the other two, which can be attributed to the decrease in Ca content in the glass with increasing Sr content. Furthermore, Sr^2+^ ions can inhibit the dissolution of Ca^2+^ ions, resulting in a continuous decrease in the concentration of Ca^2+^ ions, and ultimately leading to lower pH values in 3SMBG and 5SMBG than in 1SMBG. On the third day, a different trend emerged, with both 1SMBG and 3SMBG exhibiting a decreasing pH value, indicating that the ions in the solution had reacted to form HA. However, the high Sr content in 5SMBG delayed the formation of HA, and the glass continued to dissolve, leading to a different trend. On the seventh day, the pH values of 1SMBG and 3SMBG increased due to the continuous dissolution of the glass after the formation of HA. In contrast, 5SMBG had already formed apatite, causing a decrease in ion concentration and pH value. The changes in the pH curves of the three samples with different Sr contents were consistent with the results of weight loss analysis. Moreover, the pH values remained higher than the initial value of 7.3 after soaking for seven days, creating an environment that is conducive to the nucleation and growth of HA.

Figure 9b shows the results of ICP-OES analysis of 1S, 3S, and 5SMBG powders soaked in SBF solution. The release and variation of Sr concentrations in the samples can be observed from the figure. The ion concentration was rapidly released and increased with increasing Sr doping content on the first day of soaking. Thereafter, the concentration tended to become relatively stable or slightly decrease, showing continuous release. Notably, the concentration of Sr in 3SMBG continued to increase on the seventh day, which has not been reported in previous studies. This phenomenon may be due to the simultaneous dissolution of 3SMBG while releasing Sr ions during the formation of apatite. In contrast, 5SMBG with higher Sr doping content was generating Sr-HA, resulting in a slight decrease in ion concentration. After soaking in SBF for seven days, the Sr^2+^ concentrations of the three samples were 23.0, 59.1, and 67.1 ppm, respectively. The hydroxyapatite (HA) formation process involves three stages: initial ion dissolution from MBG surfaces, nucleation of calcium phosphate precursors, and HA crystallization. These stages are supported by FT-IR shifts in PO_4_^3−^ peaks, XRD-confirmed HA diffraction peaks, and dynamic ion release observed in ICP-OES analysis. Previous studies have shown that Sr ion concentrations between 8.7 and 87.6 ppm have a stimulating effect on osteoblasts in both in vitro and in vivo tests [35]. Thus, it can be indirectly inferred that all samples in this study can effectively improve biological responses. ICP-OES analysis confirmed that Sr was successfully incorporated into the MBG structure at levels consistent with the designed doping ratios (1–5 mol%). These quantitative data support the identification of Sr-HA phases observed in XRD and FT-IR analyses.

### 3.3. Biocompatibility Analysis of SMBG Powder

**Figure 10:**
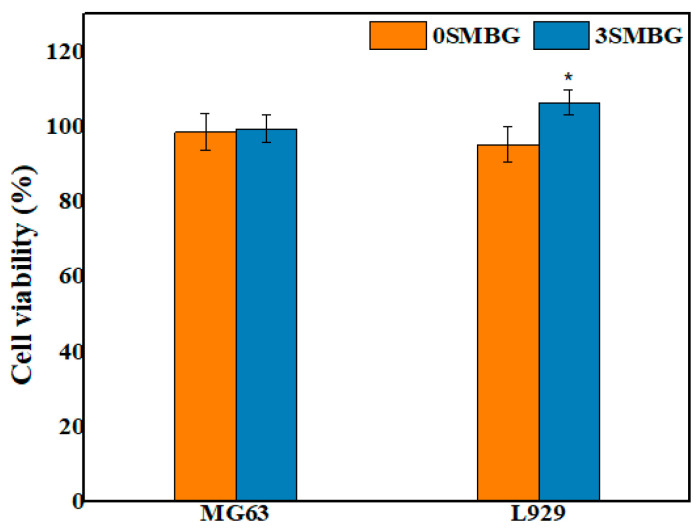
Biocompatibility analysis of 0SMBG and 3SMBG. Statistical analysis was conducted by Kruskal–Wallis H test (*: *p* < 0.05).

Biocompatibility analysis was conducted on 3SMBG, selected as the optimal parameter with Sr doping, and 0SMBG as a comparison. MG63 and L929 were cultured on both types of samples. Cell viability analysis was performed by treating the cells with a 20-fold dilution of the extract and incubating them for 24 h, followed by an Alamar Blue assay. From Figure 10, it can be observed that both 0SMBG and 3SMBG exhibited excellent biocompatibility, with cell viabilities of MG63 and L929 exceeding 95%, well above the minimum requirement of 70% specified by ISO 10993-5 standards. Furthermore, it was found that 3SMBG, with Sr doping, demonstrated even better results, particularly with L929 cells, showing a cell viability of 106.43%. This indicates that Sr doping did not induce cytotoxicity in the cells; instead, it promoted cell proliferation. Among the Sr-doped MBGs, 3SMBG demonstrated the best performance due to its optimal specific surface area (339 m^2^/g), facilitating ion exchange and hydroxyapatite nucleation. This balance between porosity and structural integrity is critical for enhancing bioactivity and achieving sustained Sr^2+^ release.

## 4. Conclusions

This study successfully synthesized MBG using the sol-gel method with P123 as a non-ionic surfactant, and developed high-silica content SMBG by substituting Sr^2+^ and Ca^2+^. BET analysis confirmed favorable specific surface areas for all SMBG samples, with 0SMBG achieving the highest value. Despite a slight reduction in surface area due to strontium incorporation, 3SMBG exhibited optimal performance with a surface area of 339 m^2^/g. SEM analysis indicated the formation of HA-like phosphatic structures after immersion in SBF for 7 days. XRD, SAED, and FT-IR analysis further validated HA formation and the presence of bioactivity-indicative functional groups such as PO_4_^3−^ and CO_3_^2−^. pH variation and ICP-OES analyses confirmed SMBG dissolution and gradual Sr^2+^ release during immersion. Prior to immersion, XRD and SAED detected amorphous or partially crystalline phases, including SrCO_3_, whereas post-immersion analyses revealed HA and Sr-substituted HA structures. Weight loss analysis and pH variation demonstrated SMBG’s propensity to dissolve and regenerate HA, with ICP-OES verifying successful strontium doping and sustained Sr^2+^ release. Comparative biocompatibility assessments showed cell viability rates exceeding 95% for both 0SMBG and 3SMBG, with superior performance observed for 3SMBG. The synergistic effects of the surfactant-induced mesoporous structure and osteogenic Sr^2+^ contribute to enhanced biocompatibility. These findings highlight the potential of SMBG, particularly 3SMBG, for bone tissue engineering applications, emphasizing their enhanced bioactivity and efficacy in promoting bone regeneration.

## Figures and Tables

**Table 1:** The composition of different MBGs.

Sample	Composition (mol %)			
	SiO_2_	CaO	P_2_O_5_	SrO
0SMBG	80	15	5	0
1SMBG	80	14	5	1
2SMBG	80	13	5	2
3SMBG	80	12	5	3
5SMBG	80	10	5	5

**Table 2:** Weight changes of 0S-5SMBG before and after immersing in SBF for 1, 3, and 7 days.

Immersion Time (Day)	Weight (g)				
	0SMBG	1SMBG	2SMBG	3SMBG	5SMBG
0	0.200 ± 0.001	0.200 ± 0.001	0.200 ± 0.001	0.200 ± 0.001	0.200 ± 0.001
1	0.190 ± 0.004	0.170 ± 0.003	0.178 ± 0.003	0.190 ± 0.004	0.178 ± 0.0032
3	0.191 ± 0.004	0.182 ± 0.004	0.193 ± 0.004	0.194 ± 0.005	0.171 ± 0.0034
7	0.188 ± 0.004	0.168 ± 0.003	0.187 ± 0.004	0.193 ± 0.004	0.182 ± 0.0039

## Data Availability

Data is contained within the article.
